# 40 Years of the Ottawa Charter: why healthy public policy still matters for health equity in Australia

**DOI:** 10.1093/heapro/daag102

**Published:** 2026-10-07

**Authors:** Carmel Williams, Samantha Thomas, Luke Wolfendon, Don Nutbeam

**Affiliations:** The Centre for Health in All Policies Research Translation, School of Public Health, Adelaide University, South Australian Health and Medical Research Institute, Adelaide, South Australia, Australia; Faculty of Health, University of Canberra, Canberra, Australian Capital Territory, Australia; Institute for Health Transformation, Faculty of Health, Deakin University, Gheringhap St, Geelong, VIC 3220, Australia; National Centre for Implementation Science, School of Medicine and Public Health, College of Health and Wellbeing, University of Newcastle, Callaghan, NSW, Australia; Sax Institute, Glebe, New South Wales, Australia; Sydney School of Public Health, University of Sydney, Sydney, NSW 2006, Australia

## Introduction

In November 2026, it will be 40 years since the first World Health Organization (WHO) International Conference on Health Promotion was held in Ottawa, Canada (World Health Organization 2026). Its significance was captured in the development of the Ottawa Charter for Health Promotion (Health Promotion International 1986), which has served as the guiding framework for the evolution of health promotion in the 40 years since publication (Thomas *et al*. 2025). This Editorial reflects on the enduring relevance of the Ottawa Charter’s concept of healthy public policy for advancing health equity. We highlight Australia’s important historical and ongoing contribution to this agenda and recognize the important role of public health research in generating the evidence, ideas, and advocacy needed to drive policy change.

## From Ottawa to Adelaide

Since the second half of the nineteenth century, public health legislation and regulation have been among the most powerful tools for protecting population health (Goodman *et al*. 2007, Heenan *et al*. 2024). Initially focused on sanitation and infectious disease control, public health policy evolved throughout the twentieth century to confront the broader social, economic, and environmental conditions that shape health. In doing so, governments recognized that health was not only produced within healthcare systems but also through the contexts in which people are born, live, work, and age. This shift cemented public policy as a central tool for protecting population health and reducing health inequalities (Carey and Crammond 2015).

The decision to hold the Ottawa Conference in Canada reflected Canada’s global leadership in public health evidence and action, driven largely by the influential ‘Lalonde Report’ (Lalonde 1974), which directly challenged dominant biomedical models of health. The Lalonde Report marked a major political shift in understandings of health. It argued that healthcare services had limited impact on overall population health compared to other determinants and highlighted that increasing medical spending alone would not significantly improve health outcomes. These ideas were extended and refined by Nancy Milio and other critical public health scholars in the lead up to the Ottawa conference. Milio reframed health and health equity as a political and structural issue, arguing that policies related to income, employment, food systems, housing, and education were fundamental drivers of these outcomes (Milio 1987). Milio is widely credited with popularizing the use of the term ‘healthy public policy’ (Milio 1987) to emphasize that improving health required coordinated approaches to health promotion across all areas and levels of government—concepts that helped to shape the intellectual foundations of the Ottawa Charter.

At the conclusion of the Ottawa conference, the Australian Government agreed to co-host a second conference focused specifically on ‘Building Healthy Public Policy’ (Department of the Prime Minister and Cabinet 1988). Aligned with the principles of the Charter, work of Australia’s Better Health Commission (1973–6) had challenged the dominance of treatment-focused healthcare and primed the Australian political system for greater investment in prevention, health promotion, and system reform (Southby 2008). Held in Adelaide in 1988, this second conference sought to operationalize the Ottawa Charter’s vision for healthy public policy (Lunnay *et al*. 2025). The resulting Adelaide Statement emphasized that all areas of government should be accountable for the health and equity impacts of their decisions, and argued that creating healthy societies required coordinated action across all sectors—not just within health departments (Health Promotion 1988). In doing so the Adelaide conference helped to lay the foundations for approaches that remain highly influential today, including health impact assessments and the concept of Health in All Policies (Health Promotion 1988).

## The role of political action in building successful healthy public policies

Australia has much to celebrate in its contribution to healthy public policy and is internationally recognized for pursuing legislative and regulatory reforms that have improved population health and reduced preventable harms. Most notably, is Australia’s global leadership in tobacco control, introducing comprehensive legislative reforms that restricted tobacco advertising, sales, and smoking in public places, alongside taxation policies designed to reduce consumption (World Health Organization 2025, Department of Health 2026). In Victoria tobacco tax reforms also enabled the establishment of the world’s first independent health promotion foundation, VicHealth which embedded prevention and health promotion within the policy architecture of government (Galbally 2000, VicHealth 2026). Australia later became the first country in the world to introduce plain packaging legislation for tobacco products despite sustained opposition and legal threats from the tobacco industry (Daube *et al*. 2012). And more recently implemented some of the world’s strictest controls on vaping and other nicotine delivery products (Freeman 2025). Importantly, this willingness to act through public policy has extended beyond tobacco control. Areas of public health action that once seemed impossible were made possible through strong public health evidence and political will—including HIV/AIDS prevention (Kirby 2022); comprehensive immunization systems (including HPV vaccinations, which have eliminated cervical cancer) (Yuill *et al*. 2026); public health campaigns to prevent skin cancer (Walker *et al*. 2022); gun control (Chapman *et al*. 2006); mandatory folic acid fortification (D'Antoine and Bower 2019); and most recently attempts to protect young people from the harms associated with social media platforms (Fardouly 2025).

These policy successes did not emerge by chance. They were supported by considerable contributions to public health research, evidence, and knowledge by Australian scientists and public health researchers and enabled by political leaders willing to confront powerful commercial, ideological, and vested interests in the pursuit of population health goals. Australian Health Ministers such as Neil Blewett, Brian Howe, and Nicola Roxon pursued ambitious and often politically contested reforms (Blewett 1996, Kirby 2009, Howe 2024). Prime Minister John Howard (1996–2007) demonstrated similar political resolve in implementing major gun control reforms following the Port Arthur massacre (Chapman *et al*. 2006). Evidence demonstrating the benefits of these and other world-leading legislative and public health policy actions have seen them adopted in other jurisdictions globally. Effective policy action also depended on strong public institutions capable of developing, implementing, and sustaining reform. Australia’s relatively strong and stable public institutions provided the institutional and bureaucratic capacity needed to translate political ambition into legislation, regulation, and implementation (Dowding and Martin 2017). Equally important was the role of the health promotion and public health profession in identifying policy priorities, generating evidence, and advocating for changes that improved health and wellbeing outcomes (Caron *et al*. 2024). Together, public health research, political leadership, capable public institutions, and a strong health promotion workforce created the conditions for Australia’s most significant healthy public policy achievements.

## Mixed success with broader whole-of-government ambitions

Whilst Australia has achieved significant success in responding to specific public health challenges, its record in implementing the broader vision of healthy public policy articulated in the Ottawa Charter has been far more uneven. Rather than isolated policy reforms, the Charter called for a whole-of-government commitment to health and equity—an agenda that later evolved into the Health in All Policies concept. This pattern has been evident across decades of reform efforts. From the Better Health Commission (Better Health Commission 1986) to the National Health Goals and Targets (Nutbeam *et al*. 1993) and Better Cities program (Neilson 2008), and the National Preventive Health Strategy (Department of Health 2021) governments have repeatedly endorsed cross-government responses to priority health issues. Yet these reforms have often lost momentum following political transition and shifting government priorities. They have also struggled to ensure effective implementation with the fidelity and research to ensure population-level improvements in health. These experiences highlight a persistent challenge for healthy public policy. While governments often endorse prevention rhetorically, sustaining long-term structural reform requires political commitment that extends beyond electoral cycles and more comprehensive and effective implementation.

At a state level, some more consistent progress can be observed, most notably with implementation of the South Australian Health in All Policies Initiative, and development of the health lens analysis process (Lawless *et al*. 2012, Baum *et al*. 2019). The South Australian HiAP initiative was designed to cast a health lens across government policies and priorities and seek opportunities for ‘win win’ policy outcomes that benefited both population heath and supported the achievement of the government’s wider sectoral priorities (Kickbusch and Buckett 2010, van Eyk *et al*. 2017). South Australian HiAP is regarded as one of the two leading examples globally on how to implement multisectoral action, and the WHO Health in All Policies 4 Pillars Model drew upon the lessons from the South Australian experience (World Health Organization 2023).

## A work in progress

While Australia has much to celebrate in its leadership on healthy public policy, progress has been uneven, politically contested, and often fragile. One less widely reported moment of the Adelaide Conference was the disruption of the opening ceremony by First Nations advocates, protesting the profound inequities experienced by Aboriginal and Torres Strait Islander peoples (Lunnay *et al*. 2025). As Prime Minister Bob Hawke took the stage to officially open the event, activists exposed a central contradiction—a nation recognized for progressive public health leadership, while Indigenous communities continued to experience drastically lower life expectancies, poor living conditions, and systemic racism (Hetzel 2000). Despite decades of concerted whole-of-government efforts to ‘Close the Gap’ in the health, social, and economic outcomes for Indigenous Australians, these inequities persist (Bond and Singh 2020, Maddox *et al*. 2025). This remains a stark reminder of the need for sustained structural reform where Indigenous ways of ‘*knowing, being, and doing can be safely applied across an array of contexts, for the benefit of all’* (Saunders *et al*. 2025).

Similarly, Australia’s public health successes have been arguably highly selective. There has been very little political resolve to tackle areas such as alcohol regulation (McInerney 2022), gambling reform (Thomas *et al*. 2023, 2026), and the climate crisis and planetary health (Xu *et al*. 2023, Nalau 2024). In these areas, governments have repeatedly retreated in the face of powerful industry lobbying, political influence, and the revolving door between politics, government, and corporate interests (Thomas *et al*. 2024). The result has been policy inertia precisely where stronger regulation has been most needed to protect health and reduce inequities. The challenge of ‘lifestyle drift’ remains one of the greatest challenges for health promotion (Robinson and Smith 2023). Governments routinely acknowledge the social and structural determinants of health, yet priorities and funding are often directed to education campaigns and behaviour change programmes. The focus on behaviour and lifestyle is attractive as it centres responsibility with the individual—and not with government or the private sector. There is a real risk that the benefits of digital technology to provide interactive, personalized support and advice will further exacerbate ‘lifestyle drift’ and divert attention from equally important structural determinants of health that require societal responses in the form of public policy (Nutbeam 2023).

At the same time, the systems needed to sustain healthy public policy are being weakened. Prevention and health promotion systems globally are consistently underfunded, with public health efforts often receiving only a fraction of the investment directed towards biomedical and clinical research or tertiary healthcare services (Latif *et al*. 2024, Shiell *et al*. 2024). This chronic underinvestment in public health threatens the independent evidence base needed to inform, defend, and advocate for healthy public policy, and is associated with a range of methodological and cultural challenges in the local political, social, and commercial contexts. For example, the flow of information and finances is increasingly impacted by commercial actors at all levels influencing the public narrative, which, in turn, affects political and investment priorities. The underinvestment of public health and health promotion research reflects broader political decisions about which forms of knowledge, expertise, and innovation governments choose to value, and weakens the capacity of governments and communities to respond to emerging threats to health equity.

## Conclusion

Australia has demonstrated leadership in healthy public policy over the past 40 years. From this experience we have learned that impactful policy reform requires sustained political commitment, strong public institutions, properly funded prevention and research systems, and a health promotion community prepared to continue advocating for structural change. As Editors in Chief, we believe public health and health promotion journals are part of the political and social infrastructure needed to defend prevention, challenge inequity, and hold governments and commercial actors accountable for health harms. Healthy public policy has always been political. Progress over the next 40 years will depend on whether governments are willing to act accordingly.

## Data Availability

Not applicable.
